# The hallmarks of neuro-immune reprogramming in cancer

**DOI:** 10.3389/fimmu.2026.1806570

**Published:** 2026-04-21

**Authors:** Jhommara Bautista, Andrés López-Cortés

**Affiliations:** Cancer Research Group (CRG), Faculty of Medicine, Universidad de Las Américas, Quito, Ecuador

**Keywords:** cancer immunity, circadian-neural orchestation, hallmarks, immunotherapy, neuro-immune reprogramming

## Abstract

Cancer immunity is commonly interpreted through tumor-centric and immune-intrinsic frameworks centered on antigenicity, immune checkpoint signaling, stromal architecture, and local immunosuppression within the tumor microenvironment. Although these models explain major determinants of immune surveillance and therapeutic response, they do not fully account for the marked heterogeneity in antitumor immunity observed across tumor types and among patients with apparently similar immunological features. Emerging evidence indicates that immune competence is also shaped by host-level regulatory systems, particularly neural and neuroendocrine pathways. Here, we propose the hallmarks of neuro–immune reprogramming in cancer as a conceptual framework to organize recurrent and analytically distinguishable modes through which neural circuits interact with tumor-, stromal-, and immune-intrinsic processes to influence antitumor immunity across its initiation, tissue access, metabolic sustainability, temporal coordination, and persistence. These hallmarks include neural calibration of innate immune set points, neurogenic control of antigen presentation and immune priming, neural gating of immune cell trafficking and tissue access, neuroendocrine constraint of immune metabolic fitness, circadian–neural orchestration of immune timing, neural imprinting of durable immunosuppressive bias, and neuro–immune–tumor circuit reinforcement. Importantly, the evidentiary maturity supporting these hallmarks is not uniform: some are supported by direct cancer-relevant mechanistic studies, whereas others currently remain best understood as cross-disciplinary inferences or testable conceptual extensions. Together, this framework positions neuro–immune regulation as an under integrated systems-level determinant of immune heterogeneity, therapeutic responsiveness, and resistance in cancer, and provides a foundation for mechanistic investigation, biomarker development, and neural-informed immunotherapeutic strategies.

## Introduction

Cancer immunity has traditionally been interpreted through tumor-centric and immune-centric frameworks (1, 2). Within these models, tumor antigenicity, mutational burden, immune checkpoint expression, stromal exclusion, and suppressive myeloid programs are viewed as the principal determinants of whether effective antitumor immunity is achieved (3, 4). This conceptual framework has enabled major therapeutic advances, most notably immune checkpoint blockade (ICB). However, it has also revealed a persistent limitation: durable immune-mediated tumor control remains uncommon across many cancer types (5, 6). Importantly, immune failure often occurs even in tumors that harbor immunogenic antigens and contain measurable immune infiltrates, indicating that antitumor responses can fail to initiate, propagate, or persist despite the apparent presence of competent immune components. These observations suggest that variation in cancer immunity cannot be fully explained by tumor-intrinsic immune evasion or immune-cell–intrinsic dysfunction alone (7, 8).

Increasing evidence from neuroimmunology, oncology, and systems physiology suggests that immune competence is also shaped by higher-order physiological regulation (9, 10). Among these influences, the nervous system occupies a central position. Neural circuits continuously integrate metabolic state, environmental cues, circadian information, and prior physiological experience, and convert these inputs into coordinated autonomic and neuroendocrine outputs that influence immune development, activation thresholds, tissue deployment, metabolic allocation, and temporal organization (9, 11, 12). In this context, neural regulation should be understood not as a substitute for established tumor-centric or immune-centric models, but as an additional layer of control that interacts with them to shape the probability, magnitude, and durability of antitumor immune responses (13, 14).

This perspective expands the interpretation of immune failure beyond local effector-stage suppression. Neural and neuroendocrine signals can act before, alongside, or in reinforcement of canonical immune pathways (15, 16). Their influence extends across multiple stages of the antitumor response, including innate sensing, antigen presentation, immune cell trafficking, metabolic fitness, and circadian timing (17–19). Consequently, tumors with broadly similar intrinsic immunogenicity may still elicit markedly different immune responses depending on host stress exposure, circadian integrity, autonomic tone, and related physiological conditions. The magnitude and direction of these effects are likely to be context dependent, varying across tumor types, tissue environments, disease stages, and individual patients (13, 20–22).

Although peripheral nerves are increasingly recognized as functional components of the tumor microenvironment (TME) and as active regulators of antitumor immunity, the available evidence remains mechanistically fragmented. Neural signals are often linked to selected immune phenotypes, such as immune exclusion, myeloid skewing, or checkpoint resistance, without an integrative framework explaining how neural regulation shapes immune initiation, spatial organization, effector durability, and therapeutic responsiveness across cellular, tissue, and systemic scales (23–25). As a result, neuroimmune findings remain compartmentalized, limiting causal integration across disciplines and constraining the systematic incorporation of neurobiological regulation into cancer immunology and immunotherapy design (26–28).

Here, we use the term hallmarks in an explicitly operational sense. A hallmark refers to a recurrent and functionally consequential mode of neuro–immune regulation that constrains a distinct organizing dimension of antitumor immunity, is supported by convergent evidence across neuroimmunology, oncology, and systems physiology, has mechanistic relevance for immune heterogeneity, therapeutic responsiveness, or resistance, and remains analytically distinguishable from the other proposed hallmarks despite their biological interdependence *in vivo* (22, 29–32). The hallmarks proposed here are therefore not intended as a closed taxonomy, but as a testable conceptual architecture for organizing an emerging field. Importantly, however, the evidentiary maturity supporting the proposed hallmarks is not uniform. Some are grounded primarily in mechanistically demonstrated cancer-relevant evidence, whereas others are better supported as cross-disciplinary inferences derived from neuroimmunology, circadian biology, and systems physiology, and a smaller subset remains more appropriately framed as forward-looking conceptual hypotheses that require direct validation in oncologic settings. Accordingly, the hallmarks proposed here should not be interpreted as equally established entities, but as analytically distinguishable regulatory dimensions that differ in evidentiary maturity while remaining useful for structuring mechanistic and translational investigation.

**Figure 1:**
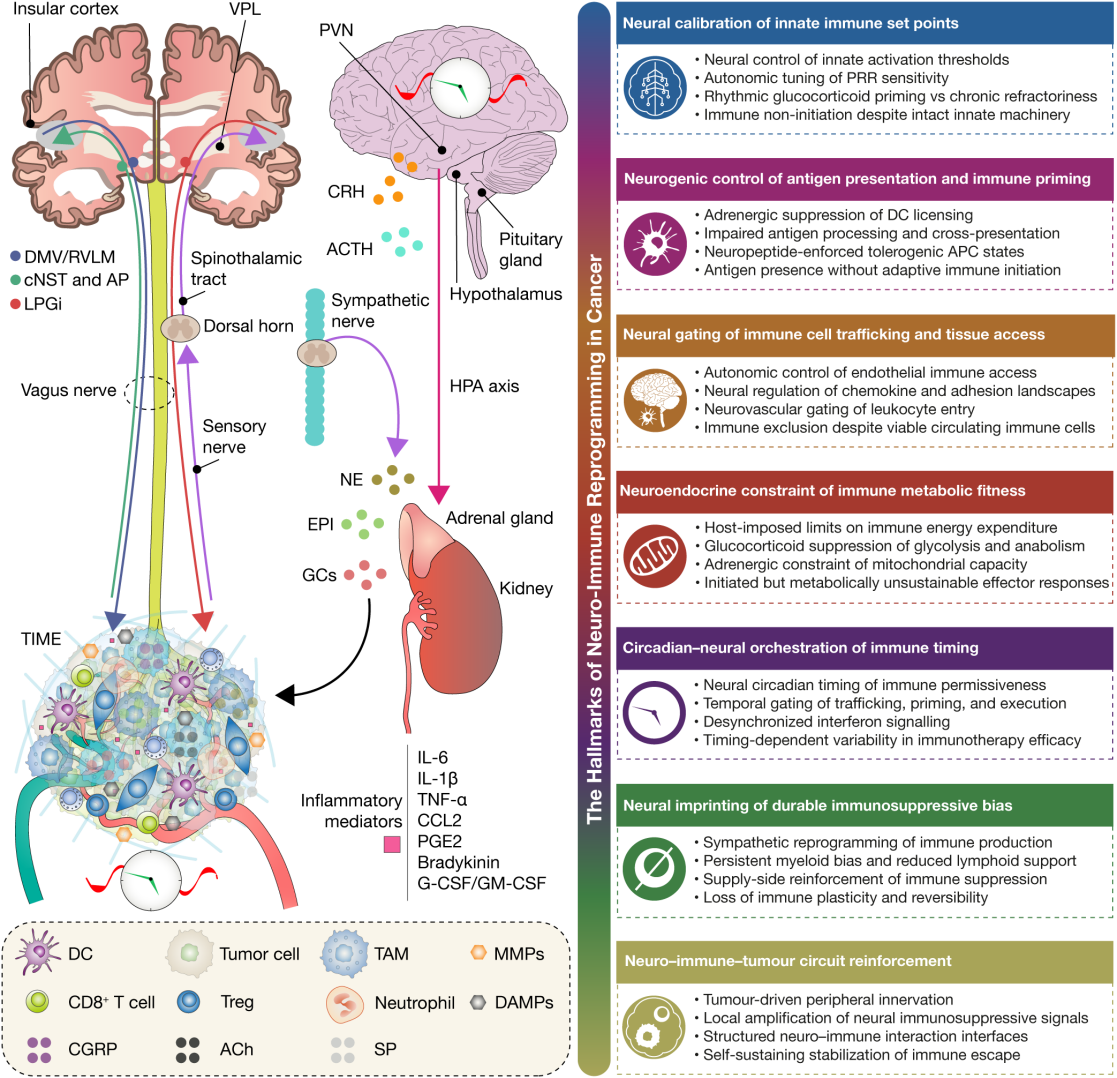
Neural circuits underlying the hallmarks of neuro–immune reprogramming in cancer. This figure illustrates how integrated neural, autonomic, and neuroendocrine circuits impose upstream control over immune competence in cancer, giving rise to the hallmarks of neuro–immune reprogramming. Sensory signals from the TIME are integrated by central neural hubs and translated into coordinated autonomic and HPA outputs that regulate immune activation thresholds, tissue access, metabolic sustainability, temporal coordination, and long-term immune plasticity. Within this architecture, neural calibration of innate immune set points emerges through autonomic and glucocorticoid control of basal immune responsiveness. Neurogenic control of antigen presentation and immune priming is mediated by adrenergic and neuropeptidergic modulation of dendritic-cell licensing, antigen processing, and cross-presentation. Neural gating of immune cell trafficking and tissue access is imposed through regulation of endothelial function, chemokine landscapes, vascular permissiveness, and lymphatic dynamics. Neuroendocrine constraint of immune metabolic fitness arises from glucocorticoid and catecholaminergic signaling that limits immune energetic investment and effector durability. Circadian–neural orchestration of immune timing defines temporal windows of immune permissiveness, coordinating trafficking, priming, and interferon programs across the day. Neural imprinting of durable immunosuppressive bias emerges through sustained neuroendocrine and sympathetic remodeling of immune production and composition, resulting in persistent myeloid bias and reduced immune plasticity. Finally, neuro–immune–tumor circuit reinforcement reflects tumor-driven innervation and reciprocal neuroimmune signaling that locally amplifies immunosuppressive programs and stabilizes immune escape. Together, the figure positions neural regulation as a hierarchical, host-level determinant of whether antitumor immunity can initiate, deploy, persist, and remain coordinated across space, metabolism, and time. TIME, tumor immune microenvironment; HPA axis, hypothalamic–pituitary–adrenal axis; GCs, glucocorticoids; NE, norepinephrine; EPI, epinephrine; ACh, acetylcholine; DC, dendritic cell; Treg, regulatory T cell; TAM, tumor-associated macrophage; MMPs, matrix metalloproteinases; DAMPs, damage-associated molecular patterns; CGRP, calcitonin gene-related peptide; SP, substance P.

In this review, we propose the hallmarks of neuro–immune reprogramming in cancer as a unifying framework for understanding how neural and neuroendocrine systems interact with tumor-, stromal-, and immune-intrinsic processes to shape antitumor immunity. These hallmarks define distinct regulatory dimensions of immune failure and immune control, encompassing neural calibration of innate immune set points (18), neurogenic control of antigen presentation and immune priming (33), neural gating of immune cell trafficking and tissue access (34), neuroendocrine constraint of immune metabolic fitness (35), circadian–neural orchestration of immune timing (36), neural imprinting of durable immunosuppressive bias (35), and neuro–immune–tumor circuit reinforcement (37) (**Figure 1**, **Table 1**). Although these hallmarks interact extensively in biological systems, each is defined here by its primary regulatory function rather than by exclusive molecular components. Throughout the review, we therefore distinguish between direct cancer-relevant mechanistic support, cross-disciplinary inference, and conceptual extension, and we align the strength of our claims with the strength of the available evidence. Rather than displacing established models of cancer immunity, this framework is intended to complement them by positioning neuro–immune regulation as an under integrated systems-level determinant of immune heterogeneity, therapeutic responsiveness, and resistance in cancer.

**Table 1:** Mechanistic framework of the hallmarks of neuro–immune reprogramming in cancer.

Hallmarks	Primary regulatory function	Key immune processes constrained	Characteristic mode of immune failure	Representative neural mediators/circuits	Predicted biomarkers/signatures
Neural calibration of innate immune set points	Sets basal thresholds for innate immune activation before tumor-associated cues are encountered	Pattern-recognition receptor sensitivity, inflammasome responsiveness, tonic cytokine tone, basal interferon readiness	Non-initiation or blunted initiation of innate immune activation despite danger signals	Glucocorticoid rhythmicity vs chronic exposure, vagal cholinergic anti-inflammatory reflex, sustained sympathetic/β-adrenergic tone	Basal interferon tone, PRR expression thresholds, inflammasome responsiveness, glucocorticoid rhythmicity, autonomic tone indices
Neurogenic control of antigen presentation and immune priming	Governs whether tumor-derived antigens are converted into productive adaptive immune initiation	Dendritic-cell maturation, costimulatory competence, IL-12 production, antigen processing, cross-presentation, cross-priming	Antigen presence without productive adaptive initiation	β-adrenergic signaling, CGRP, VIP, PACAP, neuroimmune regulation in lymphoid niches	Dendritic-cell costimulatory profiles, IL-12 deficiency, reduced cross-presentation signatures, impaired priming programs
Neural gating of immune cell trafficking and tissue access	Controls spatial immune deployment and tissue entry through vascular, lymphatic, and stromal regulation	Leukocyte rolling, adhesion, transmigration, chemokine-guided homing, lymphatic flow, tissue retention and release	Immune exclusion or maldistributed immune surveillance despite circulating immune competence	Sympathetic neurovascular units, circadian sympathetic output, CXCL12-dependent retention circuits, lymphatic autonomic regulation	Endothelial adhesion molecule rhythms, trafficking chemokine oscillations, tissue-access signatures, immune exclusion with preserved systemic immunity
Neuroendocrine constraint of immune metabolic fitness	Imposes a host-level ceiling on immune energetic investment and effector sustainability	Glycolysis, oxidative phosphorylation, mitochondrial reserve, anabolic metabolism, metabolic flexibility, myeloid bioenergetic polarization	Activated but metabolically fragile, short-lived, or incomplete effector responses	HPA-axis glucocorticoids, chronic β-adrenergic signaling, stress-hormone control of immunometabolic checkpoints	Reduced GLUT1/glycolytic programs, impaired mitochondrial fitness, low metabolic plasticity, stress-hormone–responsive immunometabolic signatures
Circadian–neural orchestration of immune timing	Synchronizes immune activation, trafficking, and responsiveness across permissive temporal windows	Rhythmic leukocyte recruitment, progenitor mobilization, interferon timing, endocrine calibration of immune responsiveness, temporal coordination of priming and infiltration	Mistimed, desynchronized, or phase-inappropriate immune responses despite preserved cellular competence	Central circadian pacemaker outputs, rhythmic sympathetic innervation, HPA rhythmicity, circadian stromal and vascular regulation	Diurnal cortisol slope, melatonin timing, actigraphy-based rhythm disruption, clock-gene phase relationships, arrhythmic trafficking or interferon signatures
Neural imprinting of durable immunosuppressive bias	Encodes persistent immunosuppressive states through chronic neural remodeling of immune supply and responsiveness	Haematopoietic stem/progenitor output, myeloid bias, extramedullary myelopoiesis, reduced dendritic-cell competence, diminished immune plasticity	Durable suppressive baseline with poor reversibility even after removal of the initiating stressor	Chronic sympathetic activation, sustained neuroendocrine stress signaling, bone marrow niche remodeling, splenic myelopoiesis	Myeloid-dominant immune composition, stress-linked haematopoietic remodeling, extramedullary myelopoiesis, low immune plasticity signatures
Neuro–immune–tumor circuit reinforcement	Establishes self-sustaining feedback loops in which tumors recruit nerves and amplify local neuroimmune suppression	Tumor innervation, dendritic-cell suppression, T-cell dysfunction/exhaustion, suppressive myeloid accumulation, vascular and stromal remodeling	Continuously regenerated immune escape maintained by tumor-amplified neural signaling	Tumor-associated sympathetic and parasympathetic innervation, β-adrenergic pathways, neurotrophins, axon-guidance cues, local neuroimmune coupling interfaces	Nerve density, neurotrophic/axon-guidance programs, adrenergic suppression signatures, innervation-associated immune exclusion or T-cell dysfunction

## Neural calibration of innate immune set points

Innate immunity in cancer is often portrayed as a reactive system that becomes engaged only after tumor-derived danger signals, inflammatory cytokines, or microbial products are detected (9). However, converging evidence from neuroimmunology supports a different model in which neural circuits continuously calibrate the basal activation thresholds of innate immune sensing before tumor-associated cues are encountered (33, 34). This model is grounded in seminal primary research using healthy rodent models, notably by Scheiermann et al., who demonstrated through surgical denervation and β-adrenoreceptor knockout mice that adrenergic nerves govern the rhythmic recruitment of leukocytes to tissues even in the absence of disease (18). In this framework, innate immunity does not operate from a neutral baseline but from a neurally imposed set point that determines whether pattern-recognition pathways remain permissive, restrained, or functionally refractory (35). Specifically, these experiments reveal that neural signals calibrate the basal expression of vascular adhesion molecules, effectively pre-conditioning the immune landscape (18, 36). Although several components of this model are strongly supported in neuroimmunology, their role as an upstream determinant of antitumor immunity remains less directly established in cancer-specific settings. This hallmark therefore concerns the threshold at which danger is detected, rather than the subsequent conversion of sensed antigen into adaptive immune initiation.

These innate immune set points arise from the integration of autonomic neurotransmission, neuroendocrine rhythms, and centrally encoded reflex architectures (36–38). Original studies using body-brain circuit mapping in mice have recently identified specific neural hubs that regulate systemic inflammatory gain (37). Within this architecture, neural tone functions as a gain-control mechanism that calibrates the sensitivity of pattern-recognition receptors (PRRs) and inflammasome responsiveness. Primary evidence for this concept includes experiments showing that exogenous acetylcholine attenuates pro-inflammatory cytokine release from lipopolysaccharide-stimulated macrophages rather than formally demonstrating a shift in toll-like receptor (TLR) ligand dose-response relationships (38). Specifically, Borovikova et al. showed that acetylcholine reduced the release of tumor necrosis factor (TNF), IL-1β, IL-6, and IL-18 from activated human macrophages, while electrical stimulation of the efferent vagus nerve suppressed TNF production *in vivo* in rodent endotoxemia (38). These findings support the concept that cholinergic neural signals can restrain the amplitude of innate inflammatory activation. However, this evidence derives from acute inflammatory and endotoxemia-related settings rather than tumor-bearing systems and should therefore be interpreted here as foundational neuroimmunology support for neurally calibrated innate immune thresholds, rather than as direct cancer-specific validation of this hallmark. Moreover, the physiological relevance of the acetylcholine concentrations used *in vitro* in relation to tumor-associated neuroimmune signaling remains uncertain.

### Neuroendocrine and autonomic calibration of innate sensitivity

Endocrine control of innate immune sensitivity illustrates this principle. Basal circadian and ultradian oscillations of glucocorticoids (GCs) tune immune responsiveness (35, 39, 40). In adrenalectomy-recovery models in rats, primary evidence has shown that at physiological concentrations, rhythmic GC signaling supports immune readiness by increasing transcriptional priming of innate sensing machinery, including PRRs and complement pathways (35, 41). By contrast, sustained or stress-level GC exposure raises activation thresholds. While these mechanisms are well-mapped in rodent physiology, human clinical data in oncology remains largely limited to correlational studies, such as associating diurnal cortisol slopes with systemic cytokine markers, leaving the direct causal link between rhythmic endocrine priming and early tumor detection in humans as a strong but still inferential extension (42, 43).

Autonomic neural circuits impose an additional layer of innate set-point control. The cholinergic anti-inflammatory reflex exemplifies a circuit-level mechanism through which the central nervous system constrains innate activation (44). In original research using rodent models of acute endotoxemia, electrical stimulation of the efferent vagus nerve or pharmacological activation of α7​ nicotinic acetylcholine receptors (α7​nAChR) on macrophages was shown to significantly reduce serum TNF levels (38, 45). Although these findings are highly influential for defining circuit-level cholinergic control of innate inflammation, they were generated in tumor-independent models of acute inflammatory challenge. Their relevance to cancer therefore lies in supporting the broader principle that neural signals can raise the threshold for productive innate activation, not in demonstrating that this mechanism has been equivalently established across tumor settings.

When cholinergic tone is elevated, early tumor-associated perturbations may fail to exceed the threshold required for productive innate activation (37, 44). Critically, a significant methodological gap remains: most evidence for this reflex stem from acute inflammatory injury, and it remains to be validated whether this circuit remains functional, or conversely becomes desensitized, during the chronic and multi-stage process of malignant progression in humans.

### Context-dependent sympathetic regulation and durable neural conditioning

Sympathetic signaling provides a complementary axis of calibration by shaping innate immune cells toward tissue-protective and regulatory states (46). Importantly, however, the immunological effects of beta-adrenergic signaling are context dependent rather than uniformly suppressive. Acute or transient noradrenergic inputs can in some settings support immune cell mobilization, redistribution, or short-term immune readiness, including effects that may favour T cell surveillance or activation (47–49). By contrast, sustained adrenergic tone, chronic stress exposure, or persistent beta-adrenergic signaling within tumor-bearing contexts more often suppresses pro-inflammatory cytokine production and promotes anti-inflammatory and resolution-associated programs, including increased interleukin-10 output and reduced responsiveness to activating cues (50–52). In peripheral tissues, such signaling can sustain macrophage phenotypes oriented toward inflammatory restraint and tissue protection rather than immune escalation. The consequences of sympathetic signaling are therefore context dependent, reflecting differences in timing, duration, receptor engagement, immune cell type, and tissue context. Importantly, these programs may emerge before overt tumor-derived cytokine conditioning and persist as a baseline property of the local immune landscape, thereby shaping how tissues interpret subsequent oncogenic danger signals (50, 51). In oncogenic settings, such preconditioned myeloid and neuroimmune states may reduce the sensitivity of innate immune sensing to early danger cues, creating conditions more permissive for malignant progression before overt adaptive immune dysfunction becomes apparent. Here, the cancer relevance is biologically plausible and partially supported by tumor-associated adrenergic immunosuppression, but the specific formulation of innate set-point calibration should still be interpreted as an integrative cancer-relevant model rather than as a uniformly demonstrated mechanism across tumor types. This distinction is particularly relevant for antitumor immunity because transient adrenergic inputs may coincide with immune mobilization, whereas sustained beta-adrenergic signaling is more consistently linked to impaired priming of tumor-specific CD8^+^ T cells and weaker downstream effector responses (21, 27, 53).

Neural control of innate immune set points is embedded within brain-integrated reflex and learning architectures rather than being limited to acute neurotransmitter or hormonal effects (45, 54). Visceral sensory inputs conveying immune-relevant information are processed by cortical and subcortical regions that shape efferent autonomic output. Repeated exposure to stress, inflammation, or tissue injury can consolidate these responses, enabling rapid redeployment of suppressive or tolerogenic programs upon subsequent challenges (55). As a result, innate immune tone can acquire a form of state dependence in which prior neural conditioning determines future responsiveness even in the absence of ongoing stimuli.

Collectively, these mechanisms redefine innate immune dysfunction in cancer as a problem of miscalibrated activation thresholds rather than simple suppression (37, 46). Neural and neuroendocrine circuits determine whether pattern-recognition receptors are sufficiently sensitive, whether tonic type I interferon programs are permissive or restrained, and whether myeloid cells are biased toward activation or tolerance at baseline (56, 57). By the time tumor-derived antigens, cytokines, and immunosuppressive factors dominate the local immune landscape, the trajectory of immune responsiveness may already be constrained by these pre-existing neurally imposed conditions (58, 59). Taken together, the available evidence supports this hallmark most strongly as a mechanistically informed cross-disciplinary inference with partial cancer relevance, and therefore as a testable framework for understanding how neural state may condition the earliest thresholds of antitumor immune responsiveness. Antitumor immunity may therefore fail not only because tumors actively suppress innate responses, but also because neural control of innate immune set points helps determine whether productive immune activation is possible at all.

## Neurogenic control of antigen presentation and immune priming

The initiation of adaptive antitumor immunity depends not only on the presence of tumor antigens, but also on whether antigen presentation and immune priming are permitted to occur within antigen-presenting cell (APC) networks (60, 61). Although this process is often framed as an intrinsic property of dendritic cells (DCs) and lymphoid architecture, evidence from neuroimmunology and cancer-relevant experimental systems indicates that neural circuits impose an additional layer of control over whether antigen encounter is translated into productive immune activation or remains immunologically silent (54). Within this framework, neural inputs function as gatekeepers of immune initiation by regulating whether antigen presentation is licensed, attenuated, or actively constrained before adaptive immune engagement begins (21, 35, 62, 63). However, the evidentiary base supporting this hallmark is heterogeneous. The strongest cancer-relevant *in vivo* support currently centers on adrenergic regulation of antigen cross-presentation, cross-priming, and the early generation of CD8^+^ T-cell responses, whereas several broader APC-regulatory and neuropeptidergic mechanisms remain supported primarily by *ex vivo*, *in vitro*, or non-tumor systems (53, 61, 64, 65). This hallmark therefore concerns the conversion of tumor-derived antigen into productive adaptive immune initiation, rather than the upstream calibration of innate sensing or the downstream deployment of effector responses.

### Neural regulation of DC licensing and antigen conversion

Neural regulation of antigen presentation operates primarily through modulation of DC maturation, costimulatory competence, and cytokine output (61). Evidence from primary research using murine and human DC cultures has shown that adrenergic signaling restricts the expression of key costimulatory molecules such as CD80 and CD86 (61, 66), while simultaneously suppressing the production of priming cytokines like IL-12 (67). APCs do not progress autonomously from antigen uptake to T cell priming, but instead require permissive contextual signals that are shaped by autonomic and neuroendocrine tone (68, 69). Catecholaminergic signaling provides a central example of this control. Noradrenergic input acting through beta-adrenergic receptors suppresses DC maturation by limiting the expression of costimulatory molecules, constraining the production of interleukin-12 and related priming cytokines, and promoting tolerogenic transcriptional programs (61–63, 67). As a result, DCs exposed to sustained adrenergic tone may acquire antigens yet fail to achieve a licensing state capable of initiating cytotoxic T cell responses (21, 35). At the same time, these DC-centered maturation and cytokine findings derive largely from reductionist experimental systems and should therefore be interpreted cautiously when extrapolated to intact tumor-bearing organisms, where stromal context, lymphoid organization, and multicellular signaling may substantially modify APC behavior.

Neural control extends beyond functional maturation to the molecular machinery of antigen processing and cross-presentation (61, 66). Original research using syngeneic mouse models of breast cancer and B-cell lymphoma has demonstrated that adrenergic signaling, driven by chronic stress or direct catecholamine exposure, impairs antigen cross-priming and the generation of functional peptide–major histocompatibility complex class I (MHC-I) complexes required for effective CD8^+^ T cell activation (16, 21, 61). This impairment reflects a failure of antigen conversion rather than a deficiency in antigen availability or lymphocyte recognition. Consequently, tumor-derived material may be present within antigen-presenting cells yet remain immunologically silent because neural inputs impose a functional block on the processes required to translate antigen capture into effective immune priming (16). This cancer-relevant *in vivo* core is further supported by work showing that β-adrenergic blockade can enhance the priming phase of antitumor CD8^+^ T cell responses and improve tumor vaccine efficacy, reinforcing the conclusion that neural signaling can act at the level of immune initiation rather than only at downstream effector stages (53). Complementing these tumor studies, *in vivo* neuroimmunology work outside cancer has also shown that sympathetic innervation can regulate both direct priming and cross-priming, supporting the broader principle that neural circuits can shape antigen-driven T cell activation in intact organisms (65).

### Neuroimmune control of priming in tissue and lymphoid niches

Neuropeptidergic signaling provides an additional and spatially refined layer of regulation. Sensory neuron-derived mediators, including calcitonin gene-related peptide, vasoactive intestinal peptide, and pituitary adenylate cyclase-activating polypeptide, directly suppress transcriptional programs required for DC activation and costimulatory competence (70). Through these mediators, local tissue innervation shapes antigen-presenting landscapes in epithelial barriers, tumor-adjacent stroma, and lymphoid niches, thereby biasing immune outcomes toward tolerance rather than activation. At the same time, the degree to which these neuropeptidergic mechanisms have been directly resolved across diverse human tumor settings remains less complete than the evidence supporting adrenergic regulation of DC function and should therefore be interpreted with appropriate caution. This sensory regulation links tissue-level neural architecture to immune decision-making in draining lymph nodes and tertiary lymphoid structures, determining whether antigen exposure leads to immune escalation or quiescence (55, 71, 72).

Neural modulation of antigen presentation is embedded within bidirectional neuroimmune circuits rather than being imposed unilaterally (9, 18). APCs express receptors for classical neurotransmitters and neuropeptides, while neurons express pattern-recognition receptors and cytokine receptors, enabling mutual sensing and feedback (71, 73). This bidirectional communication allows neural circuits to dynamically adjust priming competence in response to physiological context, stress state, and prior immune experience. Within such circuits, immune activation is not merely triggered by antigen detection but is conditionally permitted by centrally integrated information that extends beyond the TME (55). Even here, however, much of the circuitry has been defined more clearly at the level of mechanistic plausibility than through direct *in vivo* demonstration in tumor-bearing systems.

Crucially, neurogenic control of antigen presentation operates upstream of adaptive immune dysfunction and independently of immune checkpoint engagement (61). Failure at this stage does not reflect T cell exhaustion, anergy, or suppression by inhibitory receptors but rather a pre-emptive blockade of immune initiation (35, 63, 66). When DCs are prevented from achieving a licensed priming state, adaptive immunity never begins, regardless of antigen abundance or lymphocyte competence (61). This establishes a mode of immune failure characterized by non-initiation rather than suppression, in which immune responses are absent not because they are actively inhibited but because they are never triggered. In this sense, the hallmark remains conceptually distinct from downstream models of immune suppression, but its evidentiary support is strongest for an adrenergic *in vivo* core and less mature for several of its broader APC-regulatory extensions.

Neural regulation of priming also influences the architecture and functionality of lymphoid niches (18). In studies using sympathetic denervation of secondary lymphoid organs in mice, neural signals were shown to shape stromal cell behavior and cytokine gradients (e.g., CXCL12), thereby modulating the efficiency with which APCs engage naive lymphocytes (74). Through control of lymphoid microenvironments, neural circuits determine whether immune encounters are structured to support clonal expansion and differentiation or instead remain fragmented and abortive (74). These effects further reinforce the capacity of neural systems to regulate immune initiation across multiple spatial and organizational levels. Additional tumor-vaccination work suggests that sympathetic signaling can also influence the broader myeloid context in which priming occurs, although not all studies support an exclusively DC-intrinsic mechanism (26).

Collectively, these mechanisms define neurogenic control of antigen presentation and immune priming as an important upstream checkpoint in cancer immunity. Neural circuits regulate whether tumor antigens are converted into immunologically productive signals by shaping DC licensing, antigen processing, and lymphoid niche functionality (61, 62). This hallmark helps explain how tumors with comparable antigenicity and apparently intact antigen-presenting machinery can nevertheless fail to elicit adaptive immune responses. Overall, the available evidence supports this hallmark as a mixed-evidence, cancer-relevant framework: its adrenergic core is supported by direct *in vivo* studies linking neural signaling to impaired cross-presentation, cross-priming, and early CD8^+^ T cell activation in tumor settings (21, 53, 61), whereas some of its spatially extended and neuropeptidergic dimensions remain better framed as emerging extensions of that core mechanism, as much of the supporting evidence derives from VIP-, PACAP-, and CGRP-mediated regulation of DC programs in *ex vivo*, *in vitro*, or non-tumor settings (75–77). By positioning immune priming under neural influence, this hallmark reframes immune resistance in part as a failure of initiation rather than solely because of downstream suppression and supports neural regulation of antigen presentation as a plausible contributor of antitumor immune competence.

## Neural gating of immune cell trafficking and tissue access

Effective antitumor immunity requires not only immune activation but the precise spatial deployment of immune cells to relevant tissues. Immune cell trafficking is commonly attributed to tumor-derived chemokines, vascular abnormalities, and stromal barriers within the TME (4). However, accumulating evidence from neuroimmunology indicates that immune cell distribution is regulated at a higher level by neural circuits that determine where immune cells are permitted to circulate, arrest, and infiltrate under basal conditions (36, 74, 78). In this framework, spatial immune exclusion may in some cases be established before immune cells encounter tumor-derived cues and can reflect a neurally imposed geography of immune accessibility rather than a purely tumor-driven barrier (9). This model is strongly supported by mechanistic studies of neural regulation of leukocyte trafficking, circadian recruitment, and immune-cell retention, although its full translation to tumor-specific immune exclusion remains less directly demonstrated across cancer contexts. This hallmark therefore concerns physical tissue access and spatial immune distribution, rather than the upstream licensing of priming or the downstream metabolic durability of effector responses.

### Neurovascular control of immune cell entry

Neural regulation of immune trafficking is executed primarily through autonomic control of vascular and perivascular compartments. Sympathetic nerve fibers densely innervate blood vessels across tissues, forming neurovascular units in which neurotransmitter release dynamically regulates endothelial behavior (18, 54). Seminal primary research in mouse models has demonstrated that sympathetic nerves rhythmically release norepinephrine to regulate the expression of endothelial adhesion molecules, such as intercellular adhesion molecule 1 (ICAM-1) and vascular cell adhesion molecule 1 (VCAM-1), and chemokines like C-C motif chemokine ligand 2 (CCL2) (18). Through adrenergic signaling in non-haematopoietic cells, neural input controls the expression of endothelial adhesion molecules, chemokine presentation and vascular tone, thereby dictating leukocyte rolling, arrest and transmigration (36). These mechanisms operate continuously under homeostatic conditions, establishing baseline patterns of immune cell access that are independent of inflammation or tumor-derived signaling (18).

A major feature of neural control over immune trafficking is its temporal organization. Central circadian clocks entrain sympathetic outflow to peripheral tissues, generating rhythmic fluctuations in endothelial permissiveness and chemokine landscapes (18). As a result, immune cell recruitment follows robust diurnal patterns, with defined windows during which tissue entry is favored and others in which it is constrained. In rodent models of circadian misalignment, such as chronic jet-lag paradigms, the loss of rhythmic adrenergic signaling to the bone marrow and vasculature flattens these oscillations, producing inappropriate immune cell distribution despite preserved total cell numbers (18, 62). In cancer, such desynchronization may contribute to functional immune exclusion, in which immune cells remain present in circulation yet fail to access tumor-adjacent tissues during permissive windows, even in the absence of physical stromal barriers (18).

### Neural regulation of retention, redistribution, and spatial exclusion

Neural regulation of trafficking also extends beyond blood vessels to lymphatic circulation and immune cell retention within tissue niches. Sympathetic innervation of lymphatic vessels and lymphoid organs modulates lymph flow, immune cell egress, and stromal chemokine gradients that govern cellular retention (62). Original research using mouse models of hematopoietic stem cell (HSC) mobilization has shown that neural signals rhythmically regulate the expression of CXCL12 in the bone marrow niche, dictating cellular retention versus release (74). These mechanisms shape immune availability and tissue access upstream of tumor encounter and can bias immune surveillance toward or away from specific anatomical sites (78).

Stress-associated neural activation further reprograms immune cell distribution at the organismal level. Studies using chronic variable stress protocols in mice have shown that sustained sympathetic signaling activates hematopoietic stem cells, increasing myeloid cell production and redirection towards the spleen (79). This redistribution establishes persistent spatial biases in immune cell localization that can endure beyond the initiating stressor (55). In oncogenic contexts, such neural imprinting could increase immune cell abundance in non-tumor compartments while leaving tumor-adjacent tissues relatively inaccessible, thereby reinforcing immune exclusion without invoking tumor-intrinsic suppression (79).

At the tissue scale, neurovascular units function as dynamic gatekeepers that integrate neural, vascular, and immune signals. Their anatomical coupling enables rapid modulation of endothelial permeability and leukocyte transmigration, generating sharp boundaries between immune-permissive and immune-restricted zones within the same tissue (80). Unlike exclusion driven by fibrosis or extracellular matrix remodeling, neurally enforced spatial restriction is reversible and context dependent, reflecting changes in neural tone rather than structural obstruction (9, 74). This dynamic gating helps explain how immune cells can reside in proximity to tumors yet remain spatially segregated from malignant cells (55).

Sensory neurons contribute additional layers of spatial control by detecting tissue perturbations and relaying this information to central circuits that adjust autonomic output (54, 55). Through this bidirectional communication, neural systems can anticipate tissue stress and pre-emptively modulate immune trafficking, further decoupling immune cell distribution from local tumor cues (9). At present, however, the contribution of these sensory–autonomic circuits to spatial immune restriction in cancer remains more conceptually integrated than uniformly demonstrated in tumor-specific systems. Spatial immune organization therefore emerges not as a purely local response to malignancy, but as a centrally coordinated process shaped by neural state.

Collectively, these mechanisms redefine immune exclusion as a neurobiological process rather than a consequence of tumor architecture alone. Neural circuits regulate immune cell trafficking by controlling vascular access points, chemokine landscapes, lymphatic flow, and temporal windows of migration, thereby establishing spatial constraints on immune surveillance before tumor formation and independently of tumor-derived signals (18). This hallmark explains how immune cells can be viable, functional, and abundant yet systematically prevented from reaching tumor-adjacent sites (78). Overall, the available evidence supports this hallmark as mechanistically grounded in neuroimmunology and circadian trafficking biology, with indirect but biologically plausible cancer relevance that warrants direct validation in tumor-specific settings. Restoration of antitumor immunity may therefore require modulation of neural control over immune trafficking in addition to alteration of tumor chemokine expression or stromal composition, positioning neural regulation of spatial deployment as a critical determinant of immune competence in cancer.

## Neuroendocrine constraint of immune metabolic fitness

Neuroendocrine signaling exerts a dominant influence over immune cell metabolism by determining how much energetic investment immune responses are permitted to sustain (9, 54). Rather than functioning solely as immunosuppressive cues, stress hormones and neural mediators continuously regulate the metabolic programs that support immune activation, differentiation, and persistence (81). Within this framework, immune failure can occur despite intact antigen recognition, signaling, and trafficking because neural systems impose a host-level ceiling on metabolic expenditure, thereby constraining effector durability and fitness even when immune activation is successfully initiated (35). Among the hallmarks proposed here, this dimension is supported by relatively direct mechanistic evidence linking adrenergic and glucocorticoid signaling to impaired immunometabolic reprogramming and weakened antitumor effector function in cancer-relevant settings. This hallmark therefore concerns the energetic sustainability of immune function after activation has begun, rather than the upstream licensing of priming or the spatial control of tissue access.

### Neuroendocrine control of immune bioenergetic capacity

A central axis of this regulation is the hypothalamic–pituitary–adrenal (HPA) pathway, which converts neural perception of stress into systemic glucocorticoid release. Glucocorticoids regulate broad transcriptional networks in immune cells encompassing glucose uptake, lipid utilization, mitochondrial function, and redox homeostasis (82). This metabolic constraint has been characterized in rodent models of chronic stress, where elevated glucocorticoids suppress the expression of glucose transporters (e.g., GLUT1) and glycolytic enzymes, enforcing a quiescent metabolic state even in the presence of activating signals (82, 83). In T cells, this shift suppresses glycolytic flux, reduces expression of glucose transporters and glycolytic enzymes, and limits the anabolic metabolism required to sustain proliferation, cytokine production, and cytotoxic function (83). Importantly, original research in mouse models of therapy-induced immunity has demonstrated that these constraints arise independently of antigen receptor engagement and precede classical markers of immune dysfunction, indicating that neuroendocrine control of metabolism is imposed before immune exhaustion develops (21).

Adrenergic signaling provides a parallel and complementary layer of metabolic regulation (66). Sympathetic nerve fibers innervate lymphoid organs and release catecholamines that engage beta-adrenergic receptors on immune cells (36, 63). Specifically, primary research using syngeneic mouse models of lymphoma and breast cancer has demonstrated that sustained β-adrenergic signaling directly blocks the metabolic reprogramming of effector CD8^+^ T cells, leading to reduced mitochondrial functional reserve and impaired glycolytic flux during activation (21, 27). Chronic activation of these pathways interferes with the metabolic reprogramming that normally accompanies immune activation. In effector lymphocytes, sustained beta-adrenergic signaling constrains both glycolysis and oxidative phosphorylation, reduces mitochondrial mass and functional reserve, and limits the capacity to meet increased energetic demands. As a result, immune responses may be initiated but cannot be maintained, producing effector programs that are transient, energetically fragile, and functionally incomplete (21, 63).

Myeloid compartments are similarly subject to neuroendocrine metabolic reprogramming. Original studies in mouse models of anti-CTLA-4 therapy have shown that adrenergic and hormonal inputs bias macrophages toward metabolic states associated with tissue maintenance and resolution rather than sustained inflammatory or antigen-presenting activity (84). These states rely preferentially on oxidative and lipid-based metabolism and are poorly suited to support prolonged cytokine production or costimulatory function. In parallel, neural control of haematopoietic niches influences the metabolic characteristics of newly generated myeloid cells, predisposing them toward energetically constrained phenotypes before tumor encounter and shaping immune capacity at the level of immune supply (78, 79). Although this myeloid dimension is biologically well supported, its precise contribution to antitumor metabolic insufficiency remains less directly resolved than the evidence available for lymphocyte-centered adrenergic metabolic suppression.

### Metabolic timing and host-imposed energetic limits

Neuroendocrine regulation also intersects with intracellular metabolic checkpoints that integrate nutrient availability with activation state (66). Stress hormones modulate signaling nodes governing anabolic growth, mitochondrial biogenesis, and metabolic flexibility, thereby establishing a ceiling on biosynthetic investment (35). This ceiling does not abolish immune activation, but it does determine its duration and intensity, biasing immune responses toward short-lived programs incompatible with durable tumor control (21, 84). In this sense, neural and endocrine inputs function as regulators of immune energy allocation rather than simple inhibitors of immune signaling.

Temporal organization adds another layer of metabolic governance. Hormonal secretion follows circadian and ultradian rhythms that impose time-of-day-dependent variation in immune metabolic competence (85). Effector functions fluctuate accordingly, reflecting phase-specific availability of metabolic resources. When neural and endocrine rhythms are disrupted, immune activation becomes temporally misaligned with optimal metabolic windows, leading to responses that begin under energetically suboptimal conditions and decay prematurely (35). Such temporal mismatch can blunt therapeutic efficacy even when antigen presentation, trafficking, and immune signaling remain intact.

Crucially, neuroendocrine constraints on immune metabolism are distinct from tumor-induced nutrient deprivation or systemic metabolic wasting (36, 79). They operate upstream of tumor-driven metabolic competition and independently of local resource depletion. Instead, they define a host-imposed metabolic framework that governs how much energy immune responses are permitted to expend (78). In cancer, this framework can prevent immune cells from sustaining the high metabolic demands required for durable antitumor activity even in otherwise permissive immunological contexts.

Collectively, these mechanisms establish neuroendocrine regulation of immune metabolism as a fundamental determinant of effector fitness. By using primary research data from rodent models showing the suppression of glycolytic capacity and mitochondrial resilience (21, 27, 84), this hallmark positions immune metabolism not as a purely cell-autonomous property, but as a host-governed problem of resource allocation.

## Circadian–neural orchestration of immune timing

Circadian organization provides the temporal architecture within which antitumor immunity can initiate, expand, and deploy. This architecture is imposed predominantly by neural and neuroendocrine outputs rather than by immune-intrinsic clocks alone (9, 86, 87). As a result, immune competence is not constant across the 24-hour cycle but is gated into permissive and non-permissive windows by coordinated signals from the central circadian pacemaker and its autonomic and hormonal efferent. When this coordination is disrupted, immune components may remain numerically intact and molecularly competent yet operate out of phase, resulting in ineffective tumor surveillance and impaired therapeutic responsiveness (55, 78). This hallmark is strongly supported by mechanistic circadian immunology and by an expanding body of cancer-relevant evidence, although the full systems-level contribution of host circadian–neural coordination to tumor-specific immune failure remains incompletely mapped across tumor contexts. Circadian–neural desynchronization therefore constitutes a distinct failure mode of antitumor immunity in which timing, rather than immune magnitude or specificity, becomes the limiting factor (88–90). This hallmark therefore concerns temporal coordination across immune processes, rather than the individual regulation of trafficking, metabolism, or priming in isolation.

### Neural control of temporal immune coordination

Neural control of circadian immunity is executed primarily through rhythmic sympathetic innervation of vascular and stromal compartments and through oscillatory output of the HPA axis (91, 92). These signals impose daily fluctuations in endothelial adhesion molecules, chemokine gradients, and lymphatic flow, thereby defining when immune cells are permitted to circulate, extravasate, and access tissues (74). Original research in rodent models of environmental circadian disruption, such as chronic jet-lag paradigms, has demonstrated that the loss of rhythmic sympathetic signals abolishes the daily oscillations of leukocyte extravasation into peripheral tissues, resulting in an arrhythmic immune landscape (18, 62). Immune cell trafficking is therefore not merely responsive to inflammatory cues, but is also pre-patterned by neural timing signals that establish windows of tissue accessibility (18, 54). Experimental disruption of circadian organization, including jet-lag paradigms or constant light exposure, abolishes these oscillations and produces arrhythmic leukocyte recruitment despite preserved immune cell numbers (62). In cancer, such desynchronization may generate or reinforce immune-excluded landscapes before tumor recognition occurs, thereby limiting effector-cell access even in the presence of antigen and intact chemotactic machinery.

Temporal gating extends upstream to immune supply chains. Circadian neural outputs regulate haematopoietic and immune progenitor mobilization from bone marrow niches by rhythmically controlling stromal retention signals such as CXCL12 (79, 86). Evidence from mouse models of bone marrow physiology has shown that these oscillations synchronize immune cell availability and differentiation state; however, the extent to which these supply-side rhythms are preserved in cancer patients under clinical stress remains a significant area for investigation (74, 79). Circadian disruption flattens these rhythms, leading to asynchronous release of immune cells and mismatched deployment to peripheral tissues and lymphoid organs. Consequently, antitumor immune responses may fail not because priming signals are absent, but because the appropriate cellular substrates are unavailable when activation cues are delivered.

### Desynchronization, interferon timing, and therapeutic consequences

Neuroendocrine rhythms further shape immune responsiveness by dynamically calibrating basal activation thresholds and signal sensitivity (35). Circadian fluctuations in glucocorticoids tune immune receptor expression, cytokine responsiveness, and trafficking behavior across the day. These rhythms do not simply suppress immunity, but instead impose temporal structure, promoting immune activation within appropriate windows while constraining off-phase responses (93). Desynchronization of these signals uncouples immune sensing from optimal timing, generating either hypo-responsive states that fail to initiate immunity or maladaptive activation lacking spatial and functional coordination (55). Such mistimed responses are particularly detrimental in cancer, where effective immunity requires ordered transitions from innate sensing to adaptive priming and tissue infiltration (18, 94).

Type I interferon programs are especially vulnerable to circadian–neural misalignment. Interferon induction and responsiveness are temporally gated processes that depend on synchronized innate sensing and myeloid activation. Mechanistic studies in tumor-bearing mice have shown that the induction of interferon-stimulated genes (ISGs) varies significantly according to the time of day, a process that is flattened when circadian clocks are genetically or environmentally disrupted (95, 96). Neural and hormonal desynchronization shifts these programs out of phase with antigen presentation and immune cell deployment, weakening their capacity to license productive adaptive responses.

These principles have direct implications for cancer therapy. Vaccination strategies and ICB do not operate in a temporal vacuum but depend on pre-existing circadian infrastructure (93, 97). Recent seminal work in mouse models of melanoma has demonstrated that the efficacy of anti-program death-ligand 1 (PD-L1) therapy is strictly dependent on the time of administration, correlating with the circadian peak of DC-mediated T cell priming (13, 98). When immunotherapeutic interventions are delivered during non-permissive circadian phases, immune stimulation may occur without effective priming, trafficking, or effector engagement (95, 96). Conversely, circadian alignment can amplify therapeutic efficacy by synchronizing immune activation with windows of maximal cellular availability. While these mouse studies provide high-resolution mechanistic support, clinical evidence in humans relies primarily on retrospective analyses, such as the MEMOIR study, which suggests that melanoma patients receiving ICB in the morning exhibit significantly improved survival compared to those treated later in the day (99, 100).

Within this framework, resistance to immunotherapy may arise from temporal incompatibility rather than immune insufficiency. Circadian–neural desynchronization transforms antitumor immunity into a mistimed process in which immune components are present but misaligned (55). By positioning circadian control as an independent regulator of immune coordination, this hallmark establishes time as a critical dimension of cancer immunity (54, 86). Overall, the available evidence supports this hallmark as strongly grounded in circadian immunology with emerging mechanistic cancer relevance, while its full formulation as a systems-level determinant of immune timing in cancer remains partly integrative and in need of broader tumor-specific validation.

## Neural imprinting of durable immunosuppressive bias

Here, we propose neural imprinting as a durable mode of immune dysfunction in cancer in which neural circuits may encode long-lasting immunosuppressive states that persist beyond the initiating stimulus and constrain the immune system’s ability to re-enter an effective antitumor mode (21, 28, 101). Unlike transient stress-induced immunomodulation, neural imprinting represents a form of top-down immune memory in which repeated or sustained activation of sympathetic and neuroendocrine pathways reshapes immune production, composition, and responsiveness at the systems level. Once established, this imprinted state alters which immune cells are generated, how they are distributed, and how they interpret subsequent activating cues, thereby limiting the reversibility of immune dysfunction during tumor progression and therapy (16, 102). This hallmark is grounded in mechanistic evidence from chronic stress biology, haematopoietic regulation, and persistent neuroimmune state conditioning, but its formulation as a distinct cancer immunology framework remains more inferential than the hallmarks centered on priming or immunometabolic suppression. It therefore concerns durable state encoding and reduced immune plasticity over time, rather than acute regulation of priming, trafficking, metabolism, or timing.

### Chronic neural instruction of immune supply

A central mechanism underlying neural imprinting is chronic sympathetic engagement within haematopoietic niches (79). Sustained noradrenergic signaling remodels the bone marrow microenvironment by loosening stromal retention cues and increasing haematopoietic stem and progenitor cell cycling, resulting in a persistent bias toward myeloid output at the expense of lymphoid differentiation. Evidence from chronic variable stress (CVS) protocols in mice has demonstrated that sustained noradrenergic signaling remodels the bone marrow microenvironment. Specifically, seminal research by Heidt et al. revealed that sympathetic fibers release noradrenaline to down-regulate CXCL12 expression in Nestin+ stromal cells, triggering the proliferation of haematopoietic stem and progenitor cells (HSPCs) and resulting in a persistent bias toward myeloid output at the expense of lymphoid differentiation (79). Importantly, this represents reprogramming of immune supply at its source rather than transient redistribution of mature immune cells. Repeated neural input increases the likelihood that newly generated immune cells enter peripheral tissues already predisposed toward inflammatory yet tumor-permissive phenotypes (16, 62). In parallel, chronic neural activation can displace haematopoiesis to extramedullary sites such as the spleen, generating autonomous reservoirs of myeloid cell production that continue to operate long after the initiating neural stimulus has resolved. These reservoirs, characterized in mouse models of social stress and malignancy, continuously replenish suppressive innate compartments, reinforcing immune dysfunction even when bone marrow output partially normalizes (79, 103, 104).

Neural imprinting is tightly coupled to central threat appraisal and descending autonomic control rather than to immune sensing alone (35). Persistent activation of neural circuits associated with stress, threat, or chronic disease engages sympathetic efferent that repeatedly instruct immune niches and peripheral immune cells. Over time, this repeated signaling consolidates a new immune baseline characterized by sustained myeloid dominance, reduced antigen-presenting competence, and diminished lymphocyte support (79, 105). While these mechanisms are well-supported in preclinical rodent systems, clinical observations of persistent myeloid dominance in cancer patients often lack the longitudinal neural monitoring required to definitively confirm top-down imprinting in humans (79, 105).

## Reduced plasticity and persistent suppressive bias

At the cellular level, sustained adrenergic signaling imposes a unifying suppressive logic across innate and adaptive immunity (63). Chronic exposure to sympathetic neurotransmitters impairs DC maturation, migration, and costimulatory capacity, biases macrophages toward tolerogenic and wound-healing states, and dampens cytotoxic lymphocyte function (54). Although individual exposures may produce reversible inhibition, repeated signaling ensures that immune cells are continuously generated and maintained under suppressive conditions. Over time, this produces population-level effects in which immune compartments become dominated by cells intrinsically less capable of initiating or sustaining antitumor responses, conditioning the immune system to interpret antigen exposure as non-threatening and to favor tolerance over escalation (79, 102).

A defining feature of neural imprinting is reduced immune plasticity (21). In mouse models of B-cell lymphoma, the immune system loses the capacity to transition efficiently between functional modes even when inhibitory receptors are blocked, if the underlying sympathetic signaling remains active (21). Even when inhibitory receptors are blocked or tumor burden is reduced, effective priming, trafficking, and effector coordination may fail to re-emerge because the underlying immune infrastructure remains skewed (59). This provides a mechanistic explanation for why immune suppression in cancer often appears self-sustaining and poorly reversible. Rather than being maintained solely by ongoing tumor-derived signals, suppression is continuously regenerated through altered immune production and biased differentiation imposed by neural circuits (21). At present, however, this interpretation is best viewed as a systems-level model that integrates chronic neural conditioning with cancer-associated immune persistence, rather than as a uniformly demonstrated mechanism of resistance in oncology.

Neural imprinting also helps explain the persistence of immune dysfunction after removal of the original stressor. Once haematopoietic output has been reconfigured and extramedullary immune reservoirs established, a process observed to persist for weeks in murine models after the cessation of stress, the suppressive state is maintained through supply-side reinforcement (79, 103, 104). This distinguishes neural imprinting from transient stress physiology and positions it as a long-term determinant of immune competence.

Importantly, neural imprinting must be distinguished from trained immunity and other forms of immune memory. Whereas trained immunity is primarily defined by cell-intrinsic epigenetic and metabolic reprogramming of innate immune cells and their progenitors, neural imprinting describes a durable immunosuppressive state imposed and reinforced by sustained autonomic and neuroendocrine signaling at the organismal level. Neural imprinting is therefore best understood as a property of the host neuroimmune system rather than of immune cells acting autonomously (79, 106, 107). While trained immunity has been mechanistically dissected in both human volunteers (via Bacillus Calmette–Guérin (BCG) vaccination) and mice, neural imprinting as a cancer-specific failure mode remains a hypothesis that necessitates the integration of host-level neural parameters with traditional immune profiling (106, 107).

In cancer, neural imprinting provides a framework for understanding why antitumor immunity and immunotherapy frequently fail despite the presence of intact immune components. By encoding chronic immunosuppressive states upstream of tumor recognition, neural circuits can predispose the immune system to failure before immune checkpoints are engaged or tumor antigens are encountered. Immune dysfunction therefore emerges not simply because of tumor-mediated suppression, but also as the outcome of a neurally imposed constraint on immune plasticity. This hallmark predicts that effective restoration of antitumor immunity may require not only tumor-directed immune activation, but also deliberate disruption of the neural programs and immune supply architectures that maintain the imprinted suppressive state (59, 101, 108).

## Neuro–immune–tumor circuit reinforcement

Neuro–immune–tumor feedback loops describe a self-sustaining regulatory architecture in which tumors do not merely develop within a neurally regulated host, but actively remodel peripheral neural circuits and exploit neuroimmune communication to stabilize immune escape (80, 84, 101). Within these circuits, neural control of immunity is no longer an external modifier of tumor biology. Instead, it becomes a tumor-amplified program maintained through reciprocal signaling among malignant cells, stromal elements, peripheral nerves, and immune populations. At this stage, immune escape is no longer imposed transiently but continuously regenerated through coupled neural and immune reinforcement (109, 110). Among the hallmarks proposed here, this dimension is supported by some of the strongest direct cancer-relevant evidence, particularly in tumor innervation, adrenergic immunosuppression, and neural contributions to therapeutic resistance, although the full circuit-level generalization across tumor types remains integrative. This hallmark therefore concerns reciprocal circuit-level reinforcement between tumor growth, neural remodeling, and immune dysfunction, rather than one-directional neural regulation alone.

### Tumor-driven innervation and local immune reprogramming

A central initiating step in this process is tumor-driven innervation through which neural remodeling, immune dysregulation, and stromal reprogramming become coupled within tumor-permissive neuro–immune circuits (101). Evidence from rodent models of prostate and breast cancer has shown that tumors actively recruit and expand autonomic fibers. Specifically, Magnon et al. demonstrated through surgical and chemical denervation in mice that sympathetic nerves are required for early tumor phases, while parasympathetic nerves contribute to later dissemination (80). This is not a passive consequence of tissue expansion, but a tumor-instructed reconfiguration of local neural architecture that increases intratumorally availability of catecholamines and acetylcholine (36). Once this neural infrastructure is established, even basal autonomic tone becomes biologically consequential, as neurotransmitters reach immune and stromal targets rapidly and locally (18, 63, 79).

The immunological arm of the loop is reinforced by adrenergic and cholinergic signaling. Recently, Globig et al. used high-resolution T-cell profiling in mice and human cancer tissues to show that β1​-adrenergic receptor signaling in CD8^+^ T cells directly promotes an exhausted phenotype, thereby reducing the efficacy of immune checkpoint blockade (110). Sympathetic neurotransmission suppresses multiple steps required for productive antitumor immunity, including DC maturation and licensing, antigen cross-presentation, and the expansion of tumor-specific CD8+ T cells (61). Sustained beta-adrenergic signaling dampens cytotoxic T cell proliferation, IFN-γ production, and killing capacity (21). In parallel, adrenergic tone promotes the accumulation of suppressive myeloid compartments, including tumor-associated macrophages with wound-healing phenotypes (16, 111, 112).

### Neurotrophic amplification and self-reinforcing feedback

Tumors further strengthen this circuit through neurotrophic signaling. Malignant and stromal cells produce neurotrophins that drive neurite outgrowth, increasing nerve density in proximity to immune-rich stromal regions (80, 113). A defining feature of this hallmark is the emergence of neuro–immune–tumor synaptic interfaces. While these interfaces represent a compelling conceptual model of high-precision paracrine signaling, they remain an emerging extension in oncology; primary evidence for such structured microanatomical coupling comes largely from non-malignant tissue-barrier models (e.g., skin and gut) rather than established human tumor pathologies (33, 114). Within these zones, immune cells are persistently instructed, resulting in durable shifts in activation thresholds and tolerance programs.

Once established, neuro–immune–tumor circuits function as positive feedback loops. Tumor-driven innervation increases local neurotransmitter availability, suppressing antitumor immunity and facilitating tumor growth (80, 113). Critically, while these feedback loops have been mechanistically dissected in selected rodent systems (e.g., prostate and melanoma), their universal prevalence across all human cancer types remains an area where clinical validation, through high-resolution spatial imaging, is urgently needed (115). Over time, this circuit transitions from a permissive modifier into a dominant controller of tumor–immune dynamics, effectively embedding immune escape within the neurobiology of the TME (112, 115).

This framework clarifies why targeting neural signaling can reprogram tumor immunity. Experiments using pharmacological blockade of β-adrenergic signaling or genetic ablation of tumor-associated nerves in mice have shown that disrupting these circuits can remodel the immune landscape toward increased T cell infiltration (16, 76, 108). Within the feedback-loop model, neural-targeted therapies do not merely attenuate symptoms but disrupt a self-reinforcing circuit that continuously regenerates immunosuppression (117, 118). Cancer therefore emerges not solely as an immunological disease, but as a neuroimmune ecosystem capable of reinforcing its own immune permissiveness through coupled tumor-driven neurogenesis and nerve-instructed immune suppression (28, 109, 116). This hallmark thus represents a terminal state in which immune failure is not transiently imposed, but actively maintained through a self-sustaining neurobiological circuit (28, 80).

## Conclusions and future directions

Cancer immunity has traditionally been interpreted through tumor-centric and immune-centric frameworks that emphasize antigenicity, checkpoint signaling, stromal exclusion, and effector dysfunction as the principal determinants of therapeutic success or failure. Although these perspectives have enabled transformative clinical advances, they have struggled to account for a persistent observation: antitumor immunity frequently fails even when immune cells are present, antigenic targets exist, and inhibitory pathways are pharmacologically relieved. The synthesis developed in this review suggests that part of this gap reflects the incomplete integration of neural and neuroendocrine regulation into prevailing models of cancer immunity (23, 72, 108, 117).

**Figure 2:**
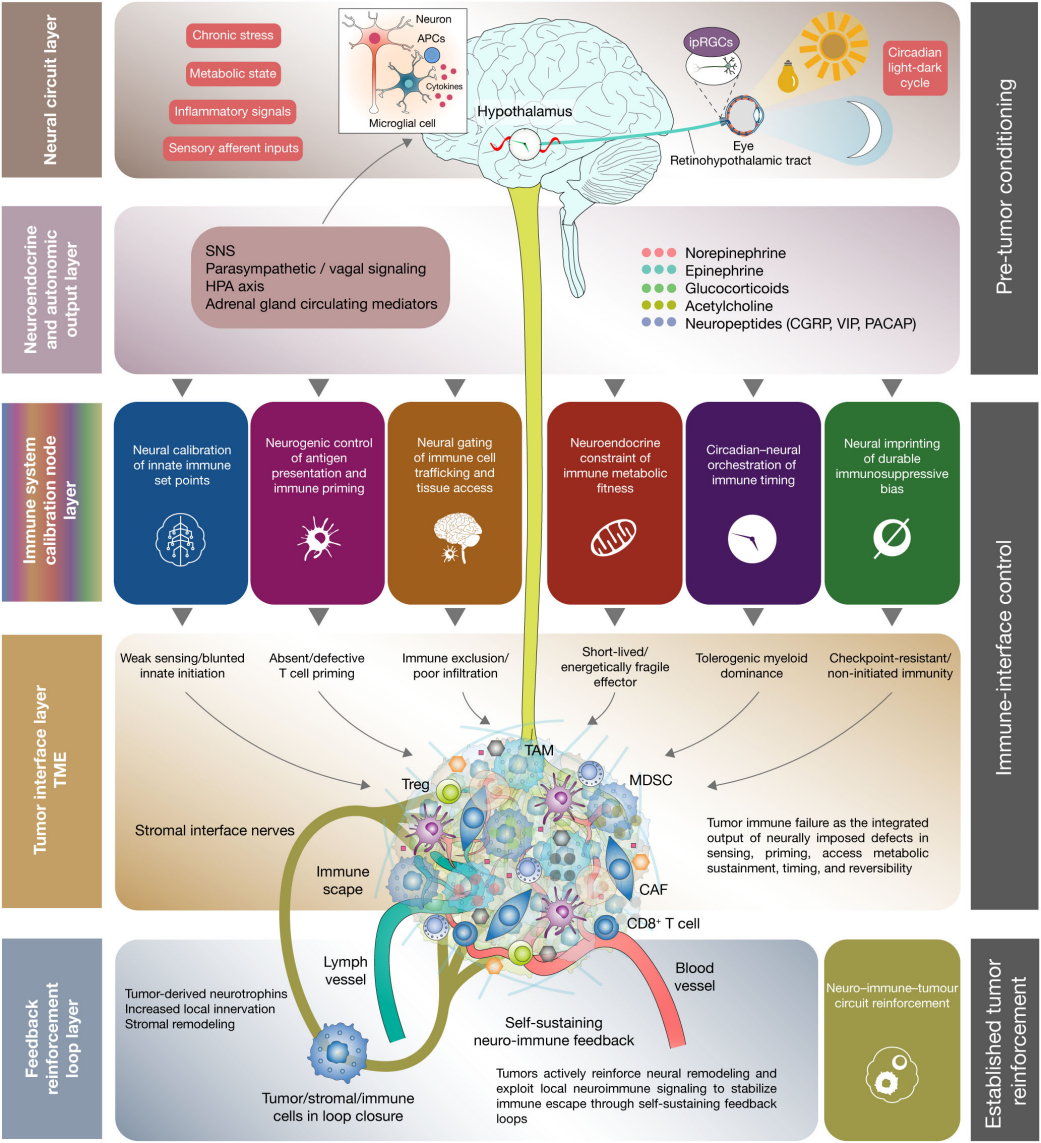
Hierarchical layered model of neuro–immune reprogramming in cancer. This figure illustrates a layered framework in which host-level neural and neuroendocrine signals shape antitumor immunity across pre-tumor conditioning, immune–tumor interface control, and established tumor reinforcement. At the neural circuit layer, central and peripheral inputs, including stress, metabolic state, inflammatory cues, sensory afferents, and circadian light–dark information, are integrated by brain regulatory hubs. These signals are translated through autonomic and neuroendocrine output pathways, including sympathetic, parasympathetic/vagal, hypothalamic–pituitary–adrenal axis, and adrenal mediators. These upstream outputs converge on immune system calibration nodes that define distinct hallmarks of neuro–immune reprogramming. At the tumor interface, these regulatory constraints manifest as weak innate sensing, defective T cell priming, immune exclusion, metabolically fragile effector function, tolerogenic myeloid dominance, and checkpoint-resistant or non-initiated immunity. At the feedback loop layer, tumor, stromal, and immune components reinforce local neural remodeling and neuroimmune signaling, generating a self-sustaining neuro–immune–tumor circuit that stabilizes immune escape. Overall, the figure positions neuro–immune reprogramming as a vertically organized systems architecture linking neural regulation to tumor immune failure and tumor-driven reinforcement. SNS, sympathetic nervous system; HPA axis, hypothalamic–pituitary–adrenal axis; CGRP, calcitonin gene-related peptide; VIP, vasoactive intestinal peptide; PACAP, pituitary adenylate cyclase-activating polypeptide; TAM, tumor-associated macrophage; MDSC, myeloid-derived suppressor cell; Treg, regulatory T cell; CAF, cancer-associated fibroblast; TME, tumor microenvironment; ipRGCs, intrinsically photosensitive retinal ganglion cells.

Across the hallmarks outlined here, the nervous system emerges not as a substitute for established tumor-centric or immune-centric explanations, but as an additional layer of systems-level regulation that shapes immune readiness before and during tumor progression (22, 118) (**Figure 2**). Neural circuits determine whether innate sensing thresholds are permissive or refractory, whether antigen presentation is licensed or silenced, whether immune cells can access tumor-adjacent tissues, how long effector programs can be metabolically sustained, and whether immune activation occurs within permissive circadian windows (11, 16, 72). In this view, immune dysfunction reflects not only local tumor-mediated suppression, but also host physiological programs that condition the timing, deployment, and durability of antitumor responses.

This framework therefore expands the interpretation of immune failure from a predominantly cell-intrinsic or tumor-localized problem to a multiscale problem of immune regulation. Tumors with comparable intrinsic immunogenicity can elicit profoundly different immune responses depending on host neural state, stress exposure, circadian organization, and autonomic tone (72). The influence of these variables is likely to be context dependent, varying across tumor types, tissue environments, disease stages, and individual patients. These constraints may help explain why ICB often fails despite tumor-infiltrating lymphocytes, intact antigen-presentation machinery, and measurable immune activation markers (23). Accordingly, effective therapy may in some settings require not only amplification of exhausted immune responses, but also relief of upstream neural constraints that shape whether immunity can be effectively initiated, deployed, and sustained.

By integrating neurobiology into cancer immunology, the hallmarks of neuro–immune reprogramming provide a unifying framework that connects innate sensing, adaptive priming, spatial deployment, metabolic fitness, temporal coordination, and immune memory under a common regulatory logic. Importantly, the evidentiary maturity of the proposed hallmarks is not uniform; while adrenergic regulation of CD8+ T cell fitness and tumor innervation are supported by high-resolution mechanistic studies in rodent models (21, 80), other hallmarks currently rely on cross-disciplinary inferences and retrospective human datasets (99, 100). This framework is intended to complement, rather than displace, established models of tumor immunity. It identifies neural–immune interfaces as mechanistically defined and potentially actionable determinants of immune heterogeneity and therapeutic responsiveness. Some dimensions, particularly those involving adrenergic regulation of antigen presentation, T cell fitness, and tumor-associated neural reinforcement, are already supported by direct cancer-relevant mechanistic studies. Others are currently better supported as cross-disciplinary inferences grounded in neuroimmunology, circadian biology, and systems physiology, while selected aspects remain more appropriately framed as forward-looking conceptual extensions requiring direct oncologic validation. Therapeutic strategies that modulate adrenergic signaling, neuroendocrine tone, or circadian alignment may therefore expose forms of immune responsiveness that remain inaccessible to immune-targeted interventions alone, particularly when combined with existing immunotherapies (33, 72, 119). Accordingly, **Table 2** was designed to distinguish their translational relevance considering their differing primary evidence bases, current cancer relevance, and near-term clinical utility.

**Table 2:** Translational and evidentiary prioritization of the hallmarks of neuro–immune reprogramming in cancer.

Hallmark	Primary evidence basis	Current cancer relevance	Main translational opportunity	Near-term clinical utility
Neural calibration of innate immune set points	Cross-disciplinary inference with partial cancer-relevant support	Likely relevant to early failure of tumor sensing and insufficient innate immune activation	Restore innate immune readiness through stress-axis normalization and autonomic recalibration	Biomarker stratification using glucocorticoid rhythmicity, autonomic tone, and basal innate-response signatures
Neurogenic control of antigen presentation and immune priming	Mechanistically demonstrated in cancer-relevant settings	Directly relevant when antigens are present but adaptive antitumor responses fail to initiate	β-adrenergic blockade or neuropeptide-targeted rescue of dendritic-cell licensing and cross-priming	Combination strategies to improve cancer vaccines, early priming, and responsiveness to immunotherapy
Neural gating of immune cell trafficking and tissue access	Mechanistically supported in neuroimmunology with indirect but plausible cancer relevance	Relevant to immune-excluded tumors despite preserved circulating immune competence	Reprogram tissue accessibility, endothelial permissiveness, and circadian trafficking windows	Stratification of immune-excluded phenotypes using trafficking, endothelial, and circadian-access signatures
Neuroendocrine constraint of immune metabolic fitness	Mechanistically demonstrated in cancer-relevant settings	Directly relevant when activated immune responses fail to persist, expand, or sustain effector function	Relieve glucocorticoid- and adrenergic-driven metabolic suppression to improve effector durability	Combination approaches integrating neural modulation with immunometabolic support
Circadian–neural orchestration of immune timing	Strong circadian immunology support with emerging cancer-relevant evidence	Highly relevant to heterogeneous immunotherapy efficacy and temporally misaligned immune coordination	Chrono-immunotherapy, circadian phase-guided scheduling, and restoration of circadian integrity	Timing-based patient stratification using cortisol slope, actigraphy, sleep–wake regularity, and circadian phase markers
Neural imprinting of durable immunosuppressive bias	Cross-disciplinary inference with partial cancer relevance and conceptual extension	Potentially relevant to chronic immune rigidity, myeloid bias, and poor reversibility of immune dysfunction	Interrupt long-term neural reinforcement and restore immune plasticity at the level of immune supply	Identification of patients with chronic stress-linked myeloid skewing, extramedullary myelopoiesis, or low immune reprogrammability
Neuro–immune–tumor circuit reinforcement	Mechanistically demonstrated in selected tumor models, with broader integrative generalization across cancers	Strongly relevant to highly innervated tumors with local neural amplification of immune escape	Tumor denervation, β-adrenergic blockade, and disruption of tumor-amplified neuroimmune loops	Patient selection using nerve density, neurotrophic programs, and combined neural–immune suppression signatures

A central translational priority now is to operationalize neuro–immune regulation through measurable biomarkers of host neural state and tumor neural architecture. Candidate biomarker strategies include tumor nerve density and spatial innervation patterns (120); neurotrophic and axon-guidance programs within tumor and stromal compartments (121); adrenergic or glucocorticoid-responsive transcriptional signatures in immune or tumor tissues (122); circulating catecholamine-related or stress-hormone profiles (123); and rhythmic parameters such as diurnal cortisol slope, melatonin timing, and clock-gene phase relationships (124). However, the clinical implementation of these markers requires careful critique, as parameters like heart-rate variability or nerve density, while robust in preclinical systems, exhibit significant inter-patient variability in human oncology that may confound their predictive value (125, 126). Initial translational efforts should prioritize clinically feasible readouts, including tumor nerve density, adrenergic or glucocorticoid-response signatures, heart-rate variability, diurnal cortisol dynamics, and actigraphy-defined circadian disruption, and determine whether these parameters stratify immunotherapy response or identify patients most likely to benefit from neural-targeted combination strategies. Rather than being treated as peripheral correlates, these measurements should be evaluated as candidate determinants of immune competence, therapeutic responsiveness, and resistance (124–126).

These considerations also have direct implications for clinical-trial design. Trials evaluating ICB, cancer vaccines, adoptive cell therapies, or combination immunotherapy could incorporate baseline neural-state stratification, including autonomic, endocrine, and circadian variables, to determine whether host neural physiology identifies subgroups with differential response probability (99, 100, 127, 128). In parallel, rational combination strategies should test whether neural-targeted interventions, such as beta-adrenergic blockade, stress-axis normalization, or circadian-restorative approaches, can relieve upstream constraints on priming, trafficking, and effector persistence when administered alongside immunotherapy (119). Such designs would allow neuro–immune regulation to be examined not only as a biological concept, but as a clinically testable axis of patient stratification and therapeutic modulation (84, 129, 130).

Temporal implementation is especially important. Circadian-based therapy scheduling should be explored as a practical translational variable rather than an ancillary consideration. Administration of vaccination, checkpoint blockade, cytokine therapy, or cell-based immunotherapy during biologically permissive windows may improve antigen presentation, leukocyte trafficking, interferon coordination, and metabolic fitness without changing drug identity or dose (100, 127). As highlighted by the transition from circadian experiments in mice to the MEMOIR retrospective study in humans, prospective trials are now essential to confirm if the observed survival benefits of morning infusions translate across diverse cancer types and individual chronotypes (99, 100). This approach may be particularly relevant in patients with circadian disruption, chronic stress, or flattened neuroendocrine rhythms, in whom temporally mistimed therapy could contribute to apparent resistance despite preserved targetability (99, 130).

Looking ahead, a central priority will be to develop experimental and clinical approaches that explicitly quantify neural regulation as a dimension of immune readiness. Current immune profiling strategies largely ignore neural tone, stress history, and circadian integrity, despite their capacity to influence immune activation thresholds and deployment. Incorporating neurophysiological, neuroendocrine, and temporal parameters into immune monitoring will be essential for explaining heterogeneity in immune surveillance, tumor progression, and therapeutic response. These efforts should be integrated with tumor-intrinsic, stromal, and immune-cell–intrinsic profiling to define when neuro–immune regulation is biologically dominant, clinically relevant, or therapeutically actionable. In this context, future biomarker frameworks should aim not only to describe neural influence retrospectively, but also to guide patient selection, treatment timing, and combination strategy design prospectively. In parallel, temporal engineering of immunotherapy, aligning vaccination, checkpoint blockade, and immune activation with permissive neural and circadian windows, represents a largely unexplored opportunity to enhance efficacy without altering drug composition.

An additional frontier lies at the intersection of neural regulation, immunity, and the microbiome (131). The gut microbiota communicates bidirectionally with the nervous system through microbial metabolites, vagal signaling, and neuroendocrine pathways, and is a well-established modulator of immune tone (90, 132). Microbiome-driven effects on stress circuits, autonomic balance, and circadian rhythms may therefore indirectly shape immune readiness by influencing neural set points upstream of immune activation (89, 133). Integrating microbiome biology into the neuro–immune framework offers a path to understanding how microbial ecosystems contribute to neural imprinting, immune calibration, and resistance to immunotherapy. It suggests that microbiome-based interventions may complement neural-targeted strategies to restore antitumor immunity (134).

Together, these insights position neuro–immune reprogramming as an important and testable dimension of cancer biology. By extending cancer immunology beyond tumor–immune interactions alone to include neural regulation of immune competence, this framework offers a broader explanation for immune heterogeneity, therapeutic responsiveness, and resistance across patients and tumor contexts. Importantly, its value does not depend on all hallmarks being equally mature, but on distinguishing which dimensions are already mechanistically actionable, which are most useful as cross-disciplinary explanatory frameworks, and which should guide future experimental validation. The next phase of cancer immunotherapy may therefore depend not only on identifying new immune targets, but also on learning how to define, measure, stratify, and therapeutically modulate the neural conditions under which effective antitumor immunity becomes possible (72, 118, 119).
